# Study of alloferon, a novel immunomodulatory antimicrobial peptide (AMP), and its analogues

**DOI:** 10.3389/fphar.2024.1359261

**Published:** 2024-02-16

**Authors:** Clara Appiah, Shitian Chen, Afia Ibnat Pori, Vladimir Retyunskiy, Chimeng Tzeng, Ye Zhao

**Affiliations:** ^1^ School of Pharmaceutical Sciences, Nanjing Tech University, Nanjing, China; ^2^ School of Pharmaceutical Sciences, Xiamen University, Xiamen, China

**Keywords:** alloferon, antimicrobial peptides, NK cell, IFN, antivirus

## Abstract

Antimicrobial peptides (AMPs) are widely distributed throughout the biosphere and represent a class of conserved peptide molecules with intrinsic antimicrobial properties. Their broad-spectrum antimicrobial activity and low risk to induce resistance have led to increased interest in AMPs as potential alternatives to traditional antibiotics. Among the AMPs, alloferon has been addressed due to its immunomodulatory properties that augment both innate and adaptive immune responses against various pathogens. Alloferon and its analogues have demonstrated pharmaceutical potential through their ability to enhance Natural Killer (NK) cell cytotoxicity and stimulate interferon (IFN) synthesis in both mouse and human models. Additionally, they have shown promise in augmenting antiviral and antitumor activities in mice. In this article, we provide a comprehensive review of the biological effects of alloferon and its analogues, incorporating our own research findings as well. These insights may contribute to a deeper understanding of the therapeutic potential of these novel AMPs.

## 1 Introduction

Since Alexander Fleming’s discovery of penicillin in 1928, a multitude of antibiotics have found extensive application in the treatment of human infections (Jenssen et al., 2006; Bahar and Ren, 2013). Typically, antibiotics are the main option to treat pathogenic microorganisms (Kościuczuk et al., 2012; Moravej et al., 2018). However, due to the recurrent and occasionally excessive use of specific antibiotics, their antibacterial efficacy has progressively diminished, leading to the emergence of drug-resistant bacteria (Rathinakumar and Wimley, 2010; Lau et al., 2018). This emphasizes the critical need to discover either new antibiotics or alternative antibacterial resources, a pursuit that has captured the attention of scientists worldwide. Since the discovery of the first antimicrobial peptide (AMP) lysozyme, more than 5,000 AMPs have been identified either through *de novo* discovery or synthesized in the laboratories (Haque et al., 2020). AMPs serve as a first line of defense against invading organisms and are an essential part of the innate immune system in a variety of species, including humans, animals, and plants, and were accordingly named host defense peptides (Brown and Hancock, 2006; Yount et al., 2006; Kościuczuk et al., 2012; Starr et al., 2018). These peptides have low molecular mass of less than 10 kDa and are known for their safety, efficiency, specificity, synthesis, and simplicity of modification (Zhang and Gallo, 2016; MJ Zeng et al.). Due to the advantages in immunomodulatory activity and the differences in their antimicrobial mechanisms compared to conventional antibiotics, clinical researchers have also shown growing interest in AMPs. AMPs can be used to combat a variety of microbes (Zhang et al., 2019; Thapa et al., 2020), including drug-resistant strains (Gennaro and Zanetti, 2000), and can be classified based on their structure, origin, biosynthesis mechanism, localization, biological function, mechanism of action, activity, and specificity (Bradshaw, 2003; Brown and Hancock, 2006; Hancock and Sahl, 2006; Harris et al., 2009). Although AMPs have a wide range of structural variations, there are several common characteristics that they share, including being relatively small (usually between 12 and 50 amino acid residues), being cationic due to the presence of numerous arginine and/or lysine residues, and having an amphipathic structure due to the presence of both hydrophobic and hydrophilic regions (Midura-Nowaczek and Markowska, 2014). AMPs have also been reported to have immunomodulatory, anti-inflammatory, and antibiofilm properties (Haney et al., 2015). Additionally, they can be used in tissue engineering and regenerative medicine and possess wound healing properties (Harman et al., 2017; Hao et al., 2022; Wang et al., 2022). Among the AMPs, alloferon has gained attention due to its immunomodulatory properties that augment both innate and adaptive immune responses against various pathogens (Chernysh et al., 2002).

Alloferon is a tridecapeptide originally isolated from a hemolymph of bacteria challenged maggots of the blow fly Calliphora vicina (Diptera) (Chernysh et al., 2002). The name of the peptide, alloferon, originates from the similarity in physiological function with interferon and origination from invertebrate (Allo). The identified amino acid sequences for alloferon are as follows: H-His-Gly-Val-Ser-Gly-His-Gly-Gln-His-Gly-Val-His-Gly-OH (alloferon I) and H-Gly-Val-Ser-Gly-His-Gly-Gln-His-Gly-Val-His-Gly-OH (alloferon II) (Chernysh et al., 2002). Alloferon has displayed anti-inflammatory and antibacterial effects, as well as immunoregulatory and immunomodulatory activities against tumors and viruses within the span of extensive research (Zhang et al., 2022). In this study, we aim to provide a comprehensive review of the biological effects and applications of alloferon and its analogues. Our focus will extend to an exploration of the antiviral and antitumor properties of alloferon, along with an assessment of potential molecular mechanisms involved.

## 2 Biological activities of alloferon

**FIGURE 1:**
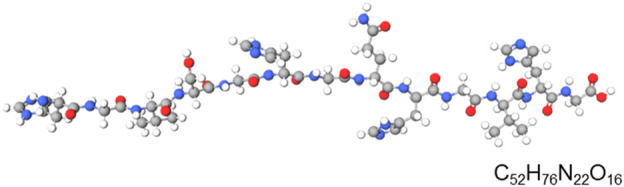
Primary structural formula of alloferon. The structural formula of alloferon with molecular formula: C_52_H_76_N_22_O_16;_ H-His-Gly-Val-Ser-Gly-His-Gly-Gln-His-Gly-Val-His-Gly-OH.

Alloferon is a linear, non-glycosylated oligopeptide with a unique amino acid sequence represented by the general formula of x1-his-gly-x2-his-gly-val-x3, where x1 is either empty or represents at least one amino acid residue, x2 is either a peptide bond or represents at least one amino acid residue, and x3 is either empty or represents at least one amino acid residue (Kowalik-Jankowska et al., 2009). The primary structure of alloferon is H-His-Gly-Val-Ser-Gly-His-Gly-Gln-His-Gly-Val-His-Gly-OH, similar to some functionally relevant proteins such as precursors of influenza virus B haemagglutinin, bovine prion protein I and II, and *Sarcophaga peregrina* antifungal protein (Kuczer et al., 2013). Figure 1 shows the primary structure of alloferon with the molecular formula C_52_H_76_N_22_O_16._

Alloferon is distinguished by being non-cytotoxic, non-immunogenic, non-mutagenic, non-carcinogenic, non-embryotoxic, and non-reproductive substance (Czarniewska et al., 2018). Alloferon can potentially be applied in treatment and prevention of various infectious and tumor diseases, where the augmentation of innate immunity, including the interferon system and naturally occurring cell-mediated cytotoxicity, plays a therapeutic role. They are used in medicine for treatment of herpes-viral infections and viral hepatitis B (Chernysh and Gordia, 2011). This section explores its diverse antiviral, antitumor, and anti-inflammatory properties, along with an examination of their possible underlying mechanisms.

### 2.1 The antiviral activity of alloferon

The effective control of viral infections and diseases is hindered by the limited availability of efficient antiviral drugs (Clercq, 2008; Lembo and Cavalli, 2010). Moreover, many existing antiviral medications exhibit a narrow spectrum of activity and can cause harm to non-infected cells. Viruses reproduce rapidly and frequently mutate, presenting a challenge in the development of new antiviral medications. Consequently, they are often rendered ineffective by viral mutations. Conversely, the process of cultivating viral cultures in laboratory conditions is frequently problematic, making it difficult to develop accurate tests for potential antiviral medications (Brown et al., 2002; Schang, 2006). In the quest for novel biologically active compounds and antimicrobial peptides, the extraction of natural compounds from plants, animals, and fungi emerges as a promising approach (Bulet and Stocklin, 2005; Kitazato et al., 2007; Slocinska et al., 2008). Natural products contain biologically active substances capable of inhibiting viruses, demonstrating antiviral action against various DNA and RNA viruses, including enteroviruses, rotaviruses, and herpes viruses (Jassim and Naji, 2003; Carriel-Gomes et al., 2007; Mohan et al., 2010).

Alloferon has demonstrated efficacy in treating infections caused by viruses such as herpesviruses, human papillomavirus and influenza virus. Herpesviruses, which can infect both vertebrate and non-vertebrate species in nature, pose a challenge due to their rapid replication and mutation rates, making the development of antiviral medications difficult (Whitley and Baron, 1996). Majewska et al. reported that alloferon I and II prevented the replication of the human herpes virus (HHV)-1 McIntyre strain in HEp-2 cells. Additionally, alloferon therapy enhanced the antiviral activity of both unstimulated and phytohaemagglutinin (PHA)-stimulated lymphocytes in infected cells (Majewska et al., 2016). In a study by Kuczer et al., a 90 μg/mL concentration of alloferon inhibited the replication of HHV-1 virus after 24 h of *in vitro* incubation (Kuczer et al., 2010). Chernysh et al. researched alloferon’s antiviral activity using a mouse model of lethal pulmonary infection with human influenza viruses A and B. Intranasal or subcutaneous administration of 25 µg of alloferon prevented mortality of the most influenza virus A-challenged animals. The injection of alloferon also stimulated the resistance of the mice to influenza virus B and prevented mortality in animals which was characterized by NK cytotoxicity of lymphocytes and IFN production (Chernysh et al., 2002). Since the potent antiviral effect of alloferon against viruses is through NK cells, it is considered an ideal candidate for combination with other antiviral medications to enhance their effectiveness (Chernysh et al., 2002). An example is showcased by Lee et al. where the combination of alloferon and zanamivir against influenza A virus (H1N1) infection *in vitro* and *in vivo* effectively prevented the development of H1N1-mediated inflammation in the lungs by inhibiting the production of inflammatory mediators and blocking the migration of inflammatory cells into lung tissue (Lee et al., 2023). The combined treatment effectively controlled viral proliferation in MDCK and A549 cells infected with H1N1. It also suppressed the production of IL-6 and MIP-1α in these cells and inhibited the activity of p38 MAPK and c-Jun, which were stimulated by H1N1 ^44^. Furthermore, the use of alloferon/zanamivir resulted in improved lung fibrosis, decreased neutrophil and macrophage infiltration in the lungs, increased survival rates in H1N1-infected mice, and prevented weight loss (Lee et al., 2023). Since the antiviral effect of alloferon was signified by blockage of inflammatory signal and mediators, it indicates alloferon’s anti-inflammatory properties contributes to its anti-viral abilities.

Another virus in the herpes family which alloferon has shown immunological effects against is the Epstein-Barr Virus (EBV) (Rakityanskaya et al., 2023). EBV is among one of the known virus types belonging to the herpes family which affects majority of people globally and continues for lifetime due to an intricate link between lytic infection and numerous types of latent infections. A recent study by Rakityanskaya et al. observed the effects of alloferon on EBV (Rakityanskaya et al., 2023). In chronic Epstein Barr Virus (CEBV), alloferon treatment significantly reduced the EBV DNA in saliva samples and greatly enhanced the cytotoxic activity of NK cells. Alloferon therapeutic effect was characterized by IFNα production and CD107a (lysome-associated membrane protein 1), an indication of the presence of cytolytic granules (Rakityanskaya et al., 2023). Alloferon has been proved applicable as antiviral and antitumor agent in the treatment of infections caused by HIV and AIDS. In regards to that is the most common neoplasm in patients with AIDS, Kaposi’s sarcoma (KS), a well-known multifocal vascular tumor that affects various organs (Brodt et al., 1997; Mehta et al., 2011). Alloferon’s action against KS was discovered through a variety of investigations conducted at different times, where follow-up research provided comprehensive details regarding the antiviral mechanisms of alloferon. ORF45 is a versatile protein which encourages infection during both the early and late stages of the viral life cycle and within hours of Kaposi sarcoma Herpesvirus (KSHV) lytic reactivation, the protein, is produced. It is crucial in encouraging the lytic cycle through a variety of mechanisms, including the suppression of the host interferon response (Atyeo and Papp, 2022). The majority of research on the function of individual KSHV interferon regulatory factors (vIRFs) also indicates that they interact with and functionally modulate cellular proteins to mitigate the cellular interferon (IFN) response and hinder various proliferative, apoptotic, and angiogenetic pathways. They have also been linked to the regulation of viral lytic replication (Koch and Schulz, 2017). Firstly, Lee et al. investigated the effect of alloferon on the regulation of lytic reactivation in KS viral infected cells, BCBL-1(*Body Cavity Based Lymphoma*) (Lee et al., 2008). The study revealed that alloferon regulated the lytic reactivation of KSHV in the infected cells by decreasing the expression level of the viral lytic activation proteins, RTA, ORF45 and vIRF2. By inhibiting the expression of these proteins, alloferon activates IFN, confirming its already known function as an enhancer of interferons. Detailed investigation by Lee et al. has further revealed that the effective antiviral activity of alloferon is by suppressing the increase of calcium influx in store-operated calcium entry (SOCE) via the downregulation of the Nuclear Factor-kappa B (NF-κB) activity (Lee et al., 2009). Additionally as studied by Lee et al. on impact of alloferon in the regulation of the life cycle of KSHV, it is reported that, alloferon exposure regulated the life cycle of KSHV by inhibiting the activation of AP-1 and enhanced the cytolytic activity of NK cells by promoting the secretion of perforin and granzyme (Lee et al., 2011). Alloferon had dual functions in its antiviral activity against KSHV by regulating the life cycle of the virus and enhancing the immune system through NK cells which was evidenced by the immune susceptibility of the BCBL-1 in the presence of alloferon.

Alloferon continues to prove its therapeutic effect in yet another infection caused by a DNA virus from the papillomaviridae family known as the Human papillomavirus (HPV). The majority of sexually active women develop the HPV as a result of early sexual initiation and frequent sexual partners. In certain instances, the infection persists and may proceed to precancerous lesions called cervical intraepithelial neoplasia (CIN) (Choi and Park, 2016). Clinically, the lesion is asymptomatic and may gradually regress or progress over time to invasive cancer (De Witte et al., 2015). The primary cause for this cervical cancer is HPV infection. Persistent HPV-associated grade I CIN is due to the imbalance of pro-inflammatory and anti-inflammatory cytokines, where anti-inflammatory signals are overexpressed and the immune system responses are suppressed (Piersma, 2011; Dugué et al., 2015; Bowden et al., 2023). Vinogradova et al. used the immunological antiviral therapy by alloferon treatment to assess the peptide’s effectiveness in grade 1 HPV-associated cervical neoplasia (Vinogradova et al., 2021). Administration of the antiviral drug alloferon (Allokin-alpha) to women suffering from CIN caused reduction in caspase-3 and caspase-9 activities, increased IL-18, and subsequent activation of IFN-γ which is an indication of the elimination of HPV (Vinogradova et al., 2021). Since IL-18 is known to activate the NK cell activation and IFN-γ is expressed after NK cell activation in viral activations, we can agree that once again the mechanism of alloferon in the treatment of CIN may be through the NK cell pathway. As known that IL-18 is a potent pro-inflammatory cytokine (Ihim et al., 2022), therefore, the observed increase in the expression of IL-18 in the presence of alloferon sustaintiates that alloferon is indeed an immune modulator and its immunomodulatory effect in viral infections is not only limited to anti-inflammation but includes pro-inflammation as well to secure a balanced immunity.

To sum up, these aforementioned investigations portray alloferon as a highly effective antiviral agent, with and NK cells and NF-κB as pathways implicated in its antiviral activity. Various studies reveal that alloferon functions as an antiviral agent in treating the viral infections by regulating the viral life cycle to prevent proliferation, controlling lytic reactivation, acting as an immunomodulator to maintain balance by inhibiting inflammatory mediators to prevent inflammation, and activating certain inflammatory signals and most of these effects were through enhancement of the NK cell cytotoxicity.

### 2.2 Antitumor activity of alloferon

Alloferon holds promise as a valuable anti-cancer agent, as its antitumor effect has been investigated using K562, one of the cell lines most sensitive to NK cytotoxicity, and the murine leukemia cell line p388 (Chernysh et al., 2002; Chernysh et al., 2012). In certain tumors, such as pancreatic cancer (PCa), a phenomenon known as “glutamine intoxication” signifies the inability of a specific cancer to survive without exogenous glutamine supplementation (Wise and Thompson, 2010; Cluntun et al., 2017). SLC6A14 is recognized for contribute to the development and vitality of tumors by facilitating glutamine uptake into cancer cells (Sniegowski et al., 2021). Due to the role of SLC6A14 in tumor progression, its inhibition may aid tumor regression and treatment. Alloferon effectively inhibited the expression of the glutamine transporter SLC6A14 in pancreatic cancer cells, thereby reducing the uptake of glutamine into the cells (Jo et al., 2022). Chernysh et al. discovered following the administration of alloferon, insignificant clusters of cancer cells were eliminated, and the growth of primary tumors visibly declined. Over the 70-day study period, a substantial percentage of animals that received subcutaneous grafts of 10^2^–10^3^ tumor cells appeared to be tumor-free (Chernysh et al., 2002).

NK cells are known to express cytotoxic granzyme B (GrB)/perforin pathway that has traditionally been regarded as the main mechanism by which cytotoxic lymphocytes eliminate allogeneic, virally infected, and/or transformed cells (Boivin et al., 2009). Bae et al. reported that alloferon increased NK cell cytotoxicity against cancer by upregulating the secretion of perforin and granzyme B (Bae et al., 2013). Furthermore, alloferon improved NK cells’ potency against cancer cells by upregulating the expression of NK-activating receptor 2B4 and increasing the production of IFN-γ, TNF-α, and granule exocytosis (Bae et al., 2013). Alloferon-1 monotherapy showed moderate tumor suppressor and tumoricidal activity comparable to low dose chemotherapy in a mouse tumor transplantation model (Chernysh et al., 2012). Moreover, both alloferon and its synthetic analogue allostatin have both been applied in combination with other chemotherapy drug *in vivo* to study their anti-cancer effects. Especially noteworthy is the ability of alloferon and allostatin, particularly the latter, to enhance the cytostatic activity of doxorubicin during P388D1 cell cloning and augment the anti-clonogenic activity of cyclophosphamide through the peptides ability to penetrate cells and bind with chromosomes (Pleskach et al., 2011). In exploring the effectiveness of nanoparticles for treating tumors, Huang et al. designed an enzyme-responsive nanoparticle, PTX-DOTAP@alloferon-1-heparin/protamine, to treat highly metastatic tumors such as melanoma. The encapsulated alloferon-1, released by ion diffusion, activated inhibited NK cells in the tumor microenvironment, contributing to the anti-tumor effect of the nanoparticle (Huang et al., 2019). Additionally, it has been reported that the mechanism of interaction between alloferon and cancer cells that involves NK cells activation in the tumor microenvironment rather than direct elimination of the tumor cell (Bae et al., 2013). Thus, the antitumor effects of alloferon are mediated through NK cell activity which is evidenced by increase in NK-cell activating receptors and cytokines, as well as cytotoxicity through cytotoxic granules.

## 3 Mechanism of action in alloferon’s antiviral and antitumor immunity

### 3.1 Alloferon and the NF-κB pathway

The majority of viral infections are characterized by NF-κB activation. NF-κB can help synchronize different immune responses necessary for infection resistance because it may stimulate the production of several proteins related to both innate and adaptive immunity. Consequently, it has been suggested that NF-κB activity during viral infection represents the host’s defense mechanism against the viral pathogen.

Through the action of pattern-recognition receptors (PRR) and cytokine-receptor interactions, the NF-κB family of transcription factors is frequently activated during viral infection, since it governs important host inflammatory and antiviral gene expression programs. Consequently, numerous viral infections encode methods to control or impede NF-κB signaling (Reus et al., 2022). When inhibitors of NF-κB (IκBs) are phosphorylated by IκB kinase (IKK) in response to an inducer, this results in the nuclear translocation of NF-κB. Once NF-κB binds to its cognate DNA, transcription of several genes involved in apoptosis, inflammation, host immunity, and cell proliferation is activated (Oeckinghaus and Ghosh, 2009). As a key transcription factor with immediate effect, NF-κB can control a variety of cellular responses, including the host’s early innate immunological response to infection. It is also linked to viral infections, septic shock syndrome, chronic inflammatory disorders, and multi-organ failure (Li and Verma, 2002). It is of no surprise that many viruses alter essential NF-κB pathway components in order to elude antiviral reactions (Zhao et al., 2015). Alloferon treatment in Nalmava cell line showed that the peptide could regulate the redox potential of infected cells by reducing the antioxidant proteins and IκBα and potentiated the immune cell antiviral effect by inducing the NF-κB pathway (Ryu et al., 2008). Based on the results of proteomic analysis, alloferon treatment demonstrated activation of the NF-κB signaling pathway, including upregulated IKK, enhanced phosphorylation of IκB kinase alpha, and decreased total IκB kinase alpha levels. Furthermore, studies also show that activation of NF-κB is involved in IFN synthesis, providing an understanding that alloferon’s ability to stimulate IFN synthesis is also linked with the NF-κB (Ryu et al., 2008). Here in this case, alloferon appears to be an activator of NF-κB pathway and thus may be one mechanism by which its antiviral effect is exerted.

However, just as viruses attempt to modulate key components of the NF-κB pathway to evade antiviral responses, they can also exploit the pathway even to promote viral infection. The NF-κB pathway serves as an attractive target for invading virus since it can impact multiple crucial events in the host cell’s life cycle, rapid activation within minutes of stimulation, and independence from protein synthesis. Numerous viruses have evolved distinct techniques to control the NF-κB pathway, including a number of human infections like herpes, hepatitis B and C viruses, influenza viruses, HIV-1, and the human T-cell leukemia virus (HTLV-1) (Sun and Cesarman, 2011). Various mechanisms by which virus activate the NF-κB pathway include binding of the viral particle to its receptor to trigger membrane-proximal signaling cascades that activate NF-κB, through viral products, such as dsRNA and viral proteins, the capsid protein VP4 and VP8 that contains a conserved TRAF-binding motif, by the accumulation of viral proteins causing an endoplasmic reticulum (ER) overload and calcium release from this cellular compartment (Santoro et al., 2003). Overall, human viral pathogens including, EBV (Sugano et al., 1997), HBV (Kim et al., 2001), CMV (cytomegalovirus) (Johnson et al., 2001), herpes simplex virus type 1 (HSV-1) (Patel et al., 1998), which are all NF-κB inducers utilize distinct strategies to control the activity of this factor. Several viruses use NF-κB activation as a tactic to prevent apoptosis and extend host cell life, which buys them more time for replication and increases the number of viral progenies.

Given that viruses can exploit the NF-κB pathway for their benefit, in certain viral infections, it might be more effective to use molecules that can inhibit this pathway for their antiviral effect. Building upon the mechanism described above, where viruses act as NF-κB inducers, we can propose the hypothesis that alloferon acts as an inhibitor of the NF-κB pathway in KSHV for its antiviral property (Lee et al., 2009). Supporting this hypothesis, Amici et al. conducted a study where the anti-inflammatory cyclopentenone prostaglandin A_1_ treated HSV infection by blocking the HSV-1 gene expression and inhibition of the NF-κB pathway (Amici et al., 2001).

**FIGURE 2:**
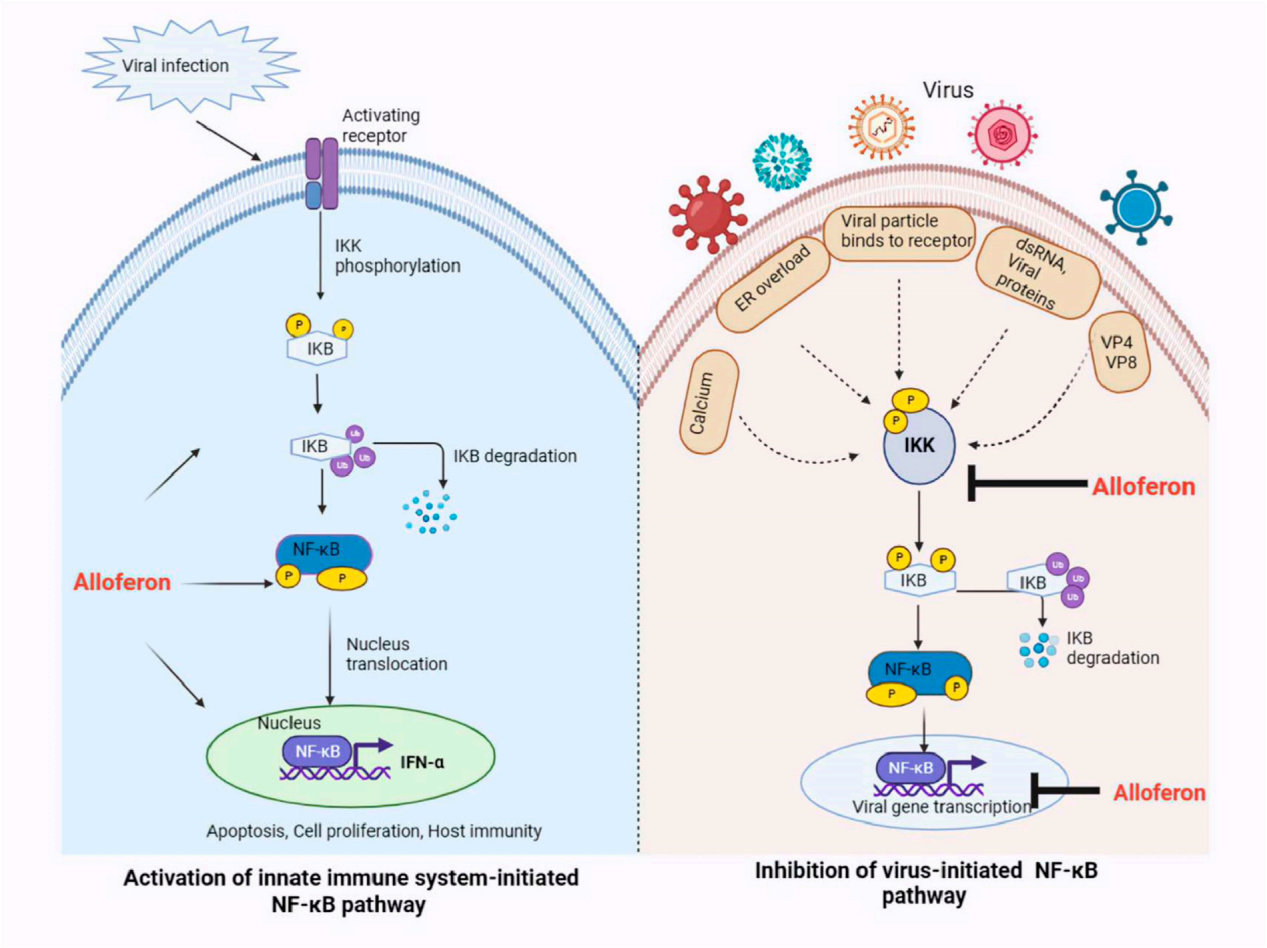
Hypothetical model for the antiviral effect of alloferon via the NF-κB pathway. Alloferon may act as an activator or inhibitor of the NF-B pathway depending on the function of the pathway in the host. During viral infections, alloferon treatment may help activate receptors and signals that boost the NF-κB pathway through IKK phosphorylation. Activation of NF-κB by alloferon causes stimulation of the IFN-α which is responsible for boosting the innate immune system to fight against the cancer or virally infected cells. On the other hand, some viruses may alter the NF-κB pathway for their benefit to induce stress responses and prevent apoptosis of the infected cells. Alloferon treatment may then act as an inhibitor of the NF-κB pathway by preventing IKK activation and blocking expression of the viral gene because some viruses possess binding sites for transcription by NF-κB.

Though conclusions reached by different studies may appear contradicting, as observed in the case of EBV infection where the virus activates NF-κB in B cells and inhibits NF-κB activation in T cells (Dreyfus et al., 1999), the only explanation may be that the particular mechanism is unique to the type of infection and conditions involved. Hence, it is plausible to propose that the antiviral effect of alloferon in CEBV infection may involve the NF-κB pathway, especially considering the implication of IFN-γ and IFN-α (Rakityanskaya et al., 2023). However, the specific mechanism, whether it entails inhibition or activation of the NF-κB pathway, remains unknown. Therefore, detailed studies are required for clarification and to elucidate the specific mechanism. In Figure 2, alloferon’s inhibitory and activating effects on the NF-κB signaling pathway are illustrated. Upon viral infection in the host, alloferon may aid in activating the NF-κB pathway as part of the host innate immune response against the virus. However, alloferon can also inhibit the NF-κB pathway, particularly when viruses activated the pathway for their own benefit.

Moreover, since both activation and inhibition of the NF-κB pathway, whether by the virus or alloferon (host), stand a chance of causing viral pathogenesis in the host, it is imperative to acknowledge the critical role of this pathway. More studies are required to gain a deeper understanding of the intricate interactions and outcomes associated with the NF-κB pathway in the context of viral infections.

### 3.2 NK cells and alloferon

NK cells play a crucial role in the human innate immune system’s defense against viruses belonging to the HPV, poxvirus, and herpesvirus families (Orange, 2002). Its antiviral defense is mostly dependent on NK cell-activating receptors, according to recent research. In summary, NK cells are triggered by viral infections through the activation of receptors (KIRS, NKps,DNAM-1,NKG2) that recognize viral products or peptide-loaded MHC molecules, or by recognizing stress-induced ligands, TLR stimulation, and cytokines (IFNs, IL-12, IL-15 and IL-18) that either directly activate NK cells for cell-mediated cytotoxicity or enhance activating receptor-mediated NK cell activation (Brandstadter and Yang, 2011). Following their activation and recruitment to the infection site, NK cells are known to utilize three primary methods to eliminate virus-infected cells: cytokine generation, granule secretion, and death receptor-mediated cytolysis (Lee et al., 2007). The NK cell mechanism indicated here seems to be extremely similar with the alloferon therapy process that we covered in this review.

Alloferon is nontoxic to normal cells and does not affect their proliferation, but has shown significant stimulatory effects on cytotoxic activity in mouse and human models. As a part of the innate immune system, NK cells are one of the first effectors at the sites of inflammation (Vivier et al., 2008), and NK cell-based immunotherapy has been extensively studied. The most commonly used NK cells in cancer immunotherapy are lymphokine-activated killer cells (LAKs) (Rosenberg et al., 1987). Natural cytotoxic lymphocytes from mouse spleen or human peripheral blood and K562 tumor cell line were targeted as effector cell, and it has been discovered that alloferon stimulates cytotoxicity in mouse spleen lymphocyte at concentrations ranging from 0.05 to 50 ng/mL (Chernysh et al., 2002). Here we discuss the specific and distinct mechanism underlying alloferon activation of NK cell.

#### 3.2.1 Alloferon enhances NK cell cytotoxicity and production of IFN

Released within target cells, perforin polymerizes to form pores, thereby facilitating the entry of granzymes into target cells. Granzymes are serine proteases that activate caspase molecules and induce apoptosis in target cells (Pardo et al., 2002; Topham and Hewitt, 2009; Martínez-Lostao et al., 2015). Perforin-dependent cytotoxicity is important for NK cell-mediated control of some tumors (Van den Broek et al., 1995; Street et al., 2001). In the context of relation of cytolitic granules secretion after NK cell activation in infected cells, Bae et al. reported that alloferon enhanced NK cell cytotoxicity against cancer by upregulation of perforin/granzyme B secretion 66. This shows that alloferon can exert antitumor effects through NK cell cytotoxicity. NK cells were identified in alloferon treatment as the peptide pharmacological target responding with immediate growth of cytotoxic activity (Ryu et al., 2008; Kowalik-Jankowska et al., 2010; Czarniewska et al., 2018). Activated NK cells secrete a wide variety of cytokines, including interferon-γ (IFN-γ) and tumor necrosis factor-α (TNF-α) (Bluman et al., 1996; Roda et al., 2006; Kwon et al., 2016). IFN-γ, one of the most potent effector cytokines, plays an important role in the antiviral, antibacterial and antitumor activities of NK cells. IFN-γ has been shown to regulate caspase, FasL, and TRAIL expression as well as promoting anti-tumor immunity (Kaplan et al., 1998). A growth in the concentrations of IFN-γ and TNF-α in the peripheral blood has been detected after alloferon treatment (Huang et al., 2019), which also suggested activation of NK cells and released IFN-γ and TNF-α *in vivo*. Moreover, in viral infections, alloferon follows similar mechanisms to enhance the immune action of NK cells and stimulate the synthesis of interferons (IFNs) during treatment (Kim et al., 2015). These mechanisms of NK cell cytotoxic activation and IFN production are closely linked, as alloferon stimulates NK cells that produce IFN upon stimulation (Biron, 1997; Ahn et al., 2001).

NK cells are involved in the development of adaptive immune responses due to their ability to produce cytokines. NK cell activation markedly increases the amount of IFN-γ and TNF-α released from the cells, reversing tumor cell-mediated immune system suppression (Selmaj and Raine, 1995; Ma et al., 2011). IFN-γ and TNF-α are biological factors capable of eliminating numerous tumor cells and activate other lymphocytes, such as T and B lymphocytes (Yu et al., 2002). Alloferon and IFN applications exibit similar pharmacological effects, centering around the synthesis of interferon and the immune response of NK cells to eliminate harmful defective cells (Bae et al., 2013). According to Rykaczewska et al., IFN-γ and TNF-α were detected in rat serum treated with alloferon. The experimental results showed that the concentrations of IFN-γ and TNF-α in rats treated with alloferon were increased (Rykaczewska-Czerwińska et al., 2011). In addition, it was demonstrated that picomolar concentrations of synthetic alloferon exert antitumor effects in mice and humans by inducing cytotoxic activity of NK cells and stimulating interferon synthesis through the activation of NF-κB (Raulet et al., 2013).

#### 3.2.2 Alloferon upregulates NK-cell activating receptors

The Natural Killer Group 2D (NKG2D) and 2B4 receptors are potentially important targets for immune surveillance, control of viral infections, pathogenesis of autoimmune diseases, and the development of malignancies. Understanding how these receptors and their ligands are regulated is important for the development of innovative immunotherapeutic strategies (Nakajima and Colonna, 2000). NKG2D and 2B4 are activation receptors that are highly expressed along with NK cells activation (Bauer et al., 1999; Bryceson et al., 2006).

The type I transmembrane protein 2B4 is present in NK cells, CD8^+^ T cells, T cells, and some subsets of CD4^+^ T cells and, upon binding to CD48, induces NK cell cytotoxicity and cytokine secretion (Davis et al., 1999; Bottino et al., 2005). In line with the function of different receptors in activating of the NK cell upon infection, Bae et al. proved that the NK cell activating receptors 2B4 was upregulated after treatment with alloferon (Bae et al., 2013). Alloferon can also enhance the cytotoxic activity of NK cells by stimulating 2B4 receptors and promoting the development of immune synapses with target cells. Therefore, it is inferred that the antitumor activity of alloferon may be mediated by the upregulation of 2B4 receptor.

NKG2D is an immune receptor that controls both innate and adoptive immune responses. In humans, NKG2D is normally expressed in all NK cells, CD8^+^ T cells, a subset of γδ T cells, and some autoreactive CD4^+^ T cells (Raulet, 2003; Zhang et al., 2015) and functions as a major activating receptor for NK cells (Bauer et al., 1999). Upon binding to its receptor, NKG2D induces cytotoxicity, cytokine secretion, and proliferation. Alloferon has been shown to alter NKG2D activity and enhance the immune response against cancer cells and viruses. Studies show that alloferon can increase the production of NKG2D in natural killer cells, thereby improving the recognition and elimination of tumor cells that produce NKG2D ligands (Bae et al., 2013). This mechanism of alloferon is consistent with the known effect of the NKG2 family receptors in activating NK cell as was mentioned earlier where NKG2D receptor is involved in recognizing stress induced ligands.

**FIGURE 3:**
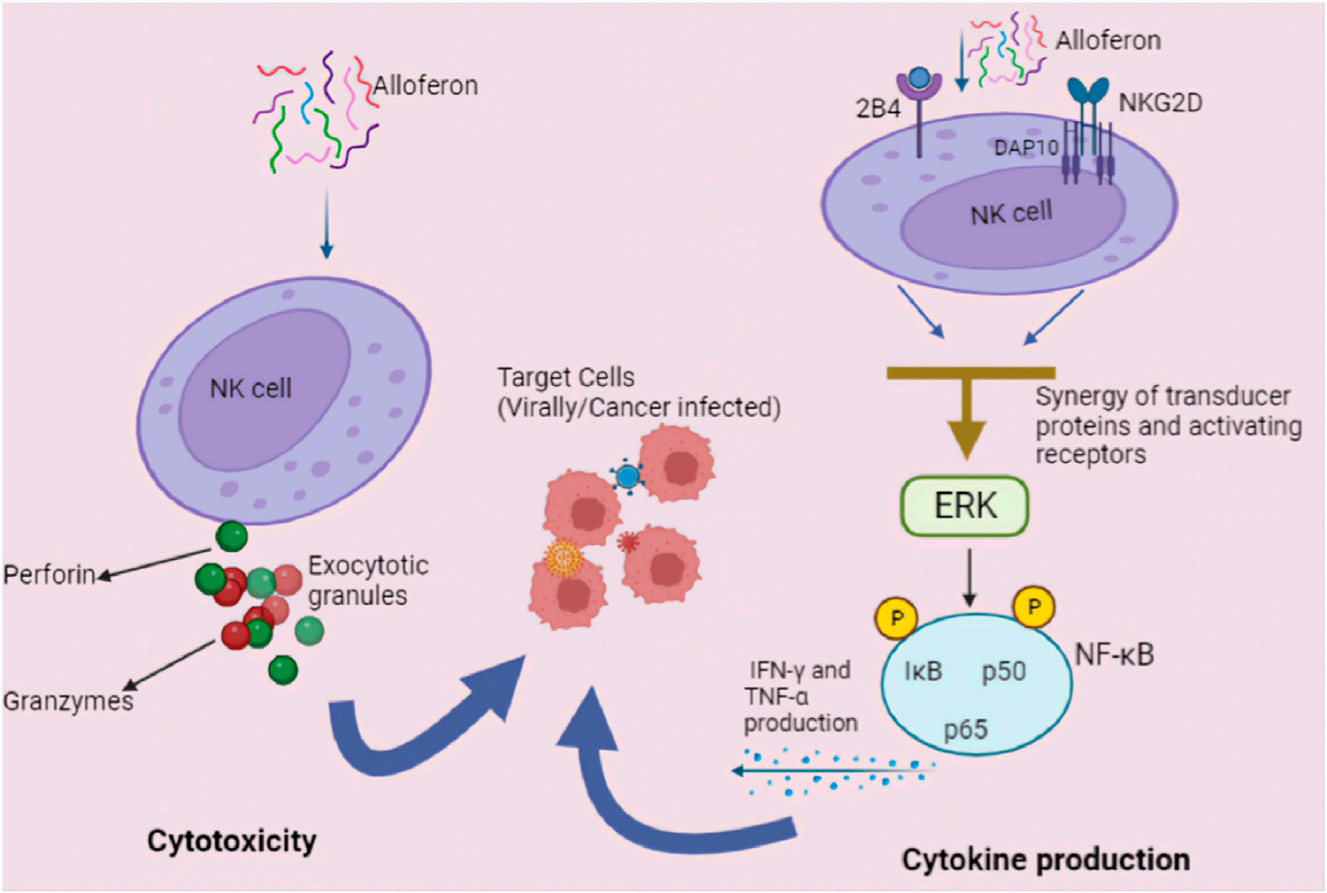
Anticancer and antiviral mechanisms of alloferon. NK cells provide early protection against cancerous and viral infected cells by producing cytokines and exerting direct cytolytic activity. Alloferon increases NK cells’ cytotoxicity following two main mechanisms: (1) Cytotoxicity via secretion of lytic/exocytotic granule. The lytic granules: perforin and granzymes functions after infected cell and NK cell interaction. Perforin causes the cell to become permeable whereas granzymes cause damage to DNA, stop the cell cycle, and destroy the nucleus when they enter the cell. (2) Increasing the production of IFN-γ and TNF-α via the NF-κB pathway. Through the generation of IFN-γ, activated NK cells generate effector activity that directly reduces host cell hospitability to the virus and can operate indirectly to prevent infection in other cells. Additionally, they recruit and activate additional effector leukocytes.

Hence in conclusion, we can speculate that mechanism of alloferon antiviral and anticancer effects involves the activation of NK cell receptors, subsequently followed up by production of cytokines and secretion of cytolytic granules for tumor and viral cells. Alloferon’s modulation of NK-cell activating receptors, such as NKG2D and 2B4, establishes a plausible pathway for its immunomodulatory and anti-tumor actions. Moreover, alloferon enhances NK cells’ ability to eliminate cancer cells and virally infected cells through two main mechanisms: 1) increasing the production of cytokines IFN-γ and TNF-α and 2) secretion of lytic/exocytotic granule. Figure 3 illustrates possible primary antiviral and anti-cancer mechanisms of alloferon through NK cells activation. However, the alloferon-NK cell pathway may be specific to different infections and various cancer diseases. Therefore, to completely comprehend the processes underpinning the effects of alloferon, futher investigation is necessary. Moreover, considering other receptors like KIRs, NKp46 and DNAM-1 have been mentioned to be involved in the process of NK cell activation, it would be greatly beneficial to delve deeply into the mechanisms of NK cell activation by alloferon to ascertain whether other receptors other than 2B4 and NKG2D are involved in this process.

### 3.3 Anti-inflammatory activity of alloferon

The anti-inflammatory and immunomodulatory properties of alloferon make it a potential therapeutic option for various inflammatory and autoimmune diseases (Hull et al., 2012). Corticosteroids are mainly *used to reduce inflammation and suppress the immune system* and are commonly used to treat asthma. However, long-term use of corticosteroids can cause side effects such as inhibition of bone growth, hypothalamic-pituitary axis inhibition, and increased opportunistic infections (Weldon, 2009). Jeon et al. found that the combination of alloferon and prednisolone for the treatment of asthma significantly diminished the numbers of eosinophils, macrophages, and neutrophils in the bronchoalveolar lavage fluid (BALF) of mice with ovalbumin (OVA)-induced asthma (Jeon et al., 2015). Alloferon prevented inflammatory cell infiltration by downregulating IL-5 and IL-17 and reduced IgG1 and IgE by inhibiting 2 T-assisted immune responses (Jeon et al., 2015). DSS-induced murine colitis is known to activate NF-κB signaling, which is important for the pathophysiology of IBD (Marrero et al., 2000; Andresen et al., 2005). The inflammatory cytokine TNF-α also increases IκB phosphorylation and activates NF-κB for transcription of many proinflammatory genes (Hayden and Ghosh). In a mouse model, alloferon was demonstrated to improve colitis induced by dextran sulfate sodium (DSS) (Kim et al., 2015). Symptoms of edema, epithelial erosion, and immune cell infiltration were alleviated by alloferon treatment, and plasma IL-6 levels in mice decreased. TNF-α treatment was shown to increase p-IκB expression and decrease IκB expression in the Colo205 colon cancer cell line (Kim et al., 2015). On the other hand, alloferon treatment inhibited TNF-α-induced degradation and phosphorylation of IκB in Colo205 colon cancer cells (Kim et al., 2015). The anti-inflammatory effect of alloferon on UVB-induced skin inflammation through the downregulation of proinflammatory cytokines has been also studied. Alloferon exhibited anti-inflammatory effect on UVB-induced skin inflammation by supressing the production of IL-1α/β, IL-6 and IL-18 in HaCaT cells after treatment (Kim et al., 2013). The activation of mitogen-activated protein kinases (MAPKs) is critical in the production of inflammatory cytokines caused by UVB irradiation (Davis, 1993; Karin and Hunter, 1995). Epidermal p38MAPK signaling is stimulated by UVB irradiation during the induction of a local pro-inflammatory response (Pfundt et al., 2001; Kim et al., 2005). Moreover, alloferon potently suppressed the activation of the UVB irradiation-induced p38MAPK and NF-κB and prevented hyperplasia in the skin of HR-1 hairless mice that were chronically exposed to UVB. Additionally, in HR-1 hairless mice, topical application of alloferon prevented epithelial thickness induced by UVB irradiation, indicating an inhibitory effect on IL-6 production 119. Furthermore, alloferon has been shown to attenuate acute inflammatory response in λ-carrageenan-induced mouse paw edema in our previous study (Zhang et al., 2022). Administration of the peptide, suppressed the expression of pro-inflammatory cytokines such as TNF-α in inflamed paw tissue, monocyte chemoattractant protein 1 (MCP1), and IL-5 (Zhang et al., 2022). In our most recent work on the effects of alloferon on estrogen deficiency-induced osteoporosis, we observed that the peptide exerted anti-inflammatory effects in a murine model of ovariectomy (OVX)-induced osteoporosis (Qiao et al., 2023). Alloferon improved estrogen deficiency-induced osteoporosis by inhibiting inflammatory signals like NLRP3 inflammasome, caspase-1, and pro-inflammatory cytokines (IL-1β and IL-18), which also confirms that the mechanism of interaction between alloferon and postmenopausal osteoporosis can potentially occur through anti-inflammatory pathway (Qiao et al., 2023).

Therefore, it can be concluded that alloferon exerts anti-inflammatory effects by modulating classical pro-inflammatory cytokine. Based on the effects and results accumulated through various studies, we can confidently endorse the already proposed mechanism of action of alloferon in treating viral and infectious diseases through the antiviral, anti-cancer, and anti-inflammatory pathways.

## 4 The structure-activity relationship of alloferon and its analogues

Antimicrobial resistance poses a serious threat to global health as it makes infections caused by bacteria, fungi and viruses more difficult to treat (Salam et al., 2023). As antibiotic resistance issue has a growing relevance, the use of alloferon and its analogues may aid the development of new therapeutic agents. Alloferon might be feasible to resolve the issue through the development of novel antimicrobial agents with either high targetability or that would improve the activities of known AMPs.

The structure of a peptide is a crucial determinant of its biological activity. In the case of AMPs like alloferon, the histidine (His) residue is believed to contribute to the antimicrobial and overall biological efficacy of the peptide. Utilizing the structure-activity relationship of peptides, innovative alloferon analogues have been developed, employing a strategy rooted in the structure-activity dependencies of proteins (Kuczer et al., 2016). The existence of hydrophilic residues at position 1 of alloferon has been found to be insignificant for the inhibitory capacity towards the replication of viruses (Kuczer et al., 2010). Antiviral identification of the analogues of alloferon showed that the first aromatic ring of the peptide chain could play a role in the expression of antiviral properties *in vitro* where the truncated analogue of alloferon without the N-terminal dipeptide His-Gly exhibited the strongest antiviral activity (Kuczer et al., 2013). Various alloferon analogues have been designed from replacement of the His residue of the peptide chain with amino acids like Arginine, Lysine, Tryptophan, Tyrosine, Alanine, etc. Other analogues, such as [des-His (Jenssen et al., 2006)]-alloferon, have also been synthesized by modifying the N or C terminals of the alloferon peptide chain through elongation or truncation (Kuczer et al., 2016). Table 1 summarizes a few of the different analogues of alloferon synthesized over the past years.

**TABLE 1:** Novel analogues of alloferon.

Alloferon analogue	Structure/amino acid sequence	Modified position	Biological activity and function	References
Native alloferon	H-His-Gly-Val-Ser-Gly-His-Gly-Gln-His-Gly-Val-His-Gly-OH	Not modified	Antiviral and antitumor effects; stimulation of NK cell activity, synthesis of interferon	Chernysh et al. (2002)
1. [Lys9]-alloferon	H-His-Gly-Val-Ser-Gly-His-Gly-Gln-Lys-Gly-Val-His-Gly-OH	Position-9 modified	2-fold increase in cytotoxic activity, increased activity against HHV-1, high biological effect in insect hemocyte	Kuczer et al. (2016)
2. [Ala9]-alloferon	H-His-Gly-Val-Ser-Gly-His-Gly-Gln-Ala-Gly-Val-His-Gly-OH	Position-9 modified	High antiviral activity towards HHV-1	Kuczer et al. (2016)
3. [Arg9]-alloferon	H-His-Gly-Val-Ser-Gly-His-Gly-Gln-Arg-Gly-Val-His-Gly-OH	Position-9 modified	Increased caspase activity against HHV-1	Kuczer et al. (2016)
4. [Phe9]-alloferon	H-His-Gly-Val-Ser-Gly-His-Gly-Gln-Phe-Gly-Val-His-Gly-OH	Position-9 modified	Pro-apoptotic activity lower than the native alloferon peptide, inhibitory activity against virus in vero cells lower than the native alloferon peptide	Kuczer et al. (2016)
5. [Phg9]-alloferon	H-His-Gly-Val-Ser-Gly-His-Gly-Gln-Phg-Gly-Val-His-Gly-OH	Position-9 modified	Highest apoptotic activity in hemocyte, inhibitory against virus in vero cells weaker than alloferon peptide	Kuczer et al. (2016)
6. [Tyr9]-alloferon	H-His-Gly-Val-Ser-Gly-His-Gly-Gln-Tyr-Gly-Val-His-Gly-OH	Position-9 modified	Reduced pro-apoptotic activity, higher antiviral effect than the native alloferon	Kuczer et al. (2016)
7. [Trp9]-alloferon	H-His-Gly-Val-Ser-Gly-His-Gly-Gln-Trp-Gly-Val-His-Gly-OH	Position-9 modified	Reduced pro-apoptotic activity, higher antiviral effect than the native alloferon	Kuczer et al. (2016)
8. [Phe(p-Cl)9]-alloferon	H-His-Gly-Val-Ser-Gly-His-Gly-Gln-Phe(p-Cl)-Gly-Val-His-Gly-OH	Position-9 modified	Strong caspase activity in hemocyte	Kuczer et al. (2016)
9. [Phe(p-OMe)9]-alloferon	H-His-Gly-Val-Ser-Gly-His-Gly-Gln-Phe(p-OMe)-Gly-Val-His-Gly-OH	Position-9 modified	Higher antiviral effect than the native alloferon	Kuczer et al. (2016)
10. [Ala12]-alloferon	H-His-Gly-Val-Ser-Gly-His-Gly-Gln-His-Gly-Val-Ala-Gly-OH	Position-12 His modified	Very low biological activity in insect hemocyte, antiviral effect lower than the native alloferon	Kuczer et al. (2016)
11. [Arg12]-alloferon	H-His-Gly-Val-Ser-Gly-His-Gly-Gln-His-Gly-Val-Arg-Gly-OH	Position-12 modified	Decreased biological activity in insect hemocyte, loss of alloferon activity	Kuczer et al. (2016)
12. [Lys12]-alloferon	H-His-Gly-Val-Ser-Gly-His-Gly-Gln-His-Gly-Val-Lys-Gly-OH	Position-12 modified	Decreased biological activity in insect hemocyte, antiviral effect lower than the native alloferon peptide	Kuczer et al. (2016)
13. [Phe12]-alloferon	H-His-Gly-Val-Ser-Gly-His-Gly-Gln-His-Gly-Val-Phe-Gly-OH	Position-12 modified	High biological activity in insect hemocyte, lower antiviral effect than the native alloferon	Kuczer et al. (2016)
14. [Phg12]-alloferon	H-His-Gly-Val-Ser-Gly-His-Gly-Gln-His-Gly-Val-Phg-Gly-OH	Position-12 modified	High biological activity in insect hemocyte, lower antiviral effect than the native alloferon	Kuczer et al. (2016)
15. [Tyr12]-alloferon	H-His-Gly-Val-Ser-Gly-His-Gly-Gln-His-Gly-Val-Tyr-Gly-OH	Position-12 modified	Decreased biological activity in hemocyte, lower antiviral effect then the native alloferon peptide	Kuczer et al. (2016)
16. [Trp12]-alloferon	H-His-Gly-Val-Ser-Gly-His-Gly-Gln-His-Gly-Val-Trp-Gly-OH	Position-12 modified	High biological activity in insect hemocyte, lower antiviral effect than the native alloferon	Kuczer et al. (2016)
17. [Phe(p-Cl)12]-alloferon	H-His-Gly-Val-Ser-Gly-His-Gly-Gln-His-Gly-Val-Phe(p-Cl)-Gly-OH	Position-12 modified	High biological activity in insect hemocyte, lower antiviral effect than the native alloferon	Kuczer et al. (2016)
18. [Phe(p-OMe)12]-alloferon	H-His-Gly-Val-Ser-Gly-His-Gly-Gln-His-Gly-Val-Phe(p-OMe)-Gly-OH	Position-12 modified	High biological activity in insect hemocyte, lower antiviral effect than the native alloferon	Kuczer et al. (2016)
19. [3–13]-Alloferon	H-Val-Ser-Gly-His-Gly-Gln-His-Gly-Val-His-Gly-OH	Sequence shortened from N-terminus (without His-Gly N-terminal amino acids)	High antiviral activity	Kuczer et al. (2013)
20. [4–13]-alloferon	H-Ser-Gly-His-Gly-Gln-His-Gly-Val-His-Gly-OH	Sequence shortened from N-terminus (without His-Gly-Val N-terminal amino acids)	Antiviral activity against HHV-1Mc in vero cells	Kuczer et al. (2013)
21. [5–13]-alloferon	H-Gly-His-Gly-Gln-His-Gly-Val-His-Gly-OH	Sequence shortened from N-terminus (without His-Gly-Val-Ser N-terminal amino acids)	Loss of antiviral activity	Kuczer et al. (2013)
22. [6–13]-alloferon	H-His-Gly-Gln-His-Gly-Val-His-Gly-OH	Sequence shortened from N-terminus (without His-Gly-Val-Ser-Gly N-terminal amino acids)	Antiviral activity against HHV-1Mc in vero cells	Kuczer et al. (2013)
23. [7–13]-alloferon	H-Gly-Gln-His-Gly-Val-His-Gly-OH	Sequence shortened from N-terminus (without His-Gly-Val-Ser-Gly-His N-terminal amino acids)	Antiviral activity against HHV-1Mc in vero cells	Kuczer et al. (2013)
24. [8–13]-alloferon	H-Gln-His-Gly-Val-His-Gly-OH	Sequence shortened from N-terminus (without His-Gly-Val-Ser-Gly-His-Gly N-terminal amino acids)	Lower antiviral and inhibitory effect than the native alloferon	Kuczer et al. (2013)
25. [9–13]-alloferon	H-His-Gly-Val-His-Gly-OH	Sequence shortened from N-terminus (without His-Gly-Val-Ser-Gly-His-Gly-Gln N-terminal amino acids)	Inactive	Kuczer et al. (2013)
26. [10–13]-alloferon	H-Gly-Val-His-Gly-OH	Sequence shortened from N-terminus (without His-Gly-Val-Ser-Gly-His-Gly-Gln-His N-terminal amino acids)	Inactive	Kuczer et al. (2013)
27. [1–11]-alloferon	H-His-Gly-Val-Ser-Gly-His-Gly-Gln-His-Gly-Val-OH	Sequence shortened from C-terminus (without His-Gly C-terminal amino acids)	Hither antiviral activity against HHV-1Mc than the native allofero	Kuczer et al. (2013)
28. [1–10]-alloferon	H-His-Gly-Val-Ser-Gly-His-Gly-Gln-His-Gly-OH	Sequence shortened from C-terminus (without Val-His-Gly C-terminal amino acids)	Lower antiviral activity against HHV-1Mc than the native allofero	Kuczer et al. (2013)
29. [1–8]-alloferon	H-His-Gly-Val-Ser-Gly-His-Gly-Gln-OH	Sequence shortened from C-terminus (without His-Gly-Val-His-Gly C-terminal amino acids)	Lower antiviral activity against HHV-1Mc than the native allofero	
30. [1–7]-alloferon	H-His-Gly-Val-Ser-Gly-His-Gly-OH	Sequence shortened from C-terminus (without Gln-His-Gly-Val-His-Gly C-terminal amino acids)	Lower antiviral activity against HHV-1Mc than the native alloferon	Kuczer et al. (2013)
31. [Phe1]-alloferon	H-Phe-Gly-Val-Ser-Gly-His-Gly-Gln-His-Gly-Val-His-Gly-OH	N-terminus modified with Phe	Inhibit replication of HHV-1Mc in Vero cells	Kuczer et al. (2013)
32. [Tyr1]-alloferon	H-Tyr-Gly-Val-Ser-Gly-His-Gly-Gln-His-Gly-Val-His-Gly-OH	N-terminus modified with Tyr	Inhibit replication of HHV-1Mc in Vero cells	Kuczer et al. (2013)
33. [Trp1]-alloferon	H-Trp-Gly-Val-Ser-Gly-His-Gly-Gln-His-Gly-Val-His-Gly-OH	N-terminus modified with Trp	Inhibit replication of HHV-1Mc in Vero cells	Kuczer et al. (2013)
34. [Phg1]-aloferon	H-Phg-Gly-Val-Ser-Gly-His-Gly-Gln-His-Gly-Val-His-Gly-OH	N-terminus modified with Phg	Inhibit replication of HHV-1Mc in Vero cells	Kuczer et al. (2013)
35. [Phe-(p-Cl)1]-alloferon	H-Phe(p-Cl)-Gly-Val-Ser-Gly-His-Gly-Gln-His-Gly-Val-His-Gly-OH	N-terminus modified with Phe(p-Cl)	Inhibit replication of HHV-1Mc in Vero cells	Kuczer et al. (2013)
36. [Phe(p-OMe)1]-alloferon	H-Phe(p-OMe)-Gly-Val-Ser-Gly-His-Gly-Gln-His-Gly-Val-His-Gly-OH	N-terminus modified with Phe(p-OMe)	Inhibit replication of HHV-1Mc in Vero cells	Kuczer et al. (2013)
37. [Lys1]-alloferon	H-Lys-Gly-Val-Ser-Gly-His-Gly-Gln-His-Gly-Val-His-Gly-OH	Position-1 His substituted with Lys	Higher activity against herpes and coxsackie viruses, than the native alloferon peptide	Kuczer et al. (2011)
38. [Arg1]-alloferon	H-Arg-Gly-Val-Ser-Gly-His-Gly-Gln-His-Gly-Val-His-Gly-OH	Position-1 His substituted with Arg	Active against herpes and coxsackie viruses	Kuczer et al. (2011)
39. [Ala1]-alloferon	H-Ala-Gly-Val-Ser-Gly-His-Gly-Gln-His-Gly-Val-His-Gly-OH	Position-1 His substituted with Ala	Complete loss of antiviral activity against herpes and coxsackie viruses	Kuczer et al. (2011)
40. [des-His1]-alloferon	H-Gly-Val-Ser-Gly-His-Gly-Gln-His-Gly-Val-His-Gly-OH	Shortened N-terminus	Active against herpes and coxsackie viruses	Kuczer et al. (2011)
41. Allostatine	H-His-Gly-Val-Ser-Gly-His-Gly-Gln-His-Gly-Thr-His-Gly-OH	Position-11 Val substituted with Thr	Higher tumorostatic effect then the native alloferon peptide	Chernysh and Kozuharova (2013)

The structure-activity relationships of novel derivatives of the insect peptide demonstrate that the substitution of different positions in alloferon peptide chain with other amino acids results in analogues with differing activities (Kuczer et al., 2011). Alloferon analogues: [Lys9]-, [Phg9]-, [Phe(p-Cl)9]-, [Phe(p-oMe)9]-, [Phe12]-, [Phg12]-, [Trp12]-, [Phe(p-Cl)12]-, and [Phe(p-oMe)12]- alloferons have all been studied to have great apoptotic activity towards the hemocytes of *Tenebrio molitor* (Kuczer et al., 2011). Further study has revealed that the modifications: [Ala9]-, [Arg9]-, and [Lys9]- alloferons possess increased antiviral activity against Human Herpes Virus (HHV)-1, with particular analogues exerting 2-fold biological activities compared to the initial alloferon molecule (Kuczer et al., 2016). Similarly, alloferon analogues that were generated by replacing the His residue at position 1 have also shown to inhibit the replication of HHV-1 in Vero cells (Kuczer et al., 2010). Kuczer et al. evaluated other analogues of alloferon through truncation of the peptide chain and modification of the terminus and found that the new peptides hinder the replication of DNA and RNA viruses (HHV-1 and Coxsackievirus) *in vitro* at lower or equal doses compared to the native alloferon peptide (Kuczer et al., 2011; Kuczer et al., 2013). Another structural analogue of alloferon, referred to as allostatine, demonstrates potent antitumor activity. Allostatine exhibits a prevailing tumoristatic effect over alloferon-1 in naïve animals where allostatine administration enhanced the vaccination efficacy (Chernysh and Kozuharova, 2013). Its notable antitumor activity positions it as a potential adjuvant in cancer immunotherapy. Czarniewska et al. conducted a study on the long-term immune effects of three structural analogues of alloferon: [Phe(p-NH_2_)1]-, [Phe(p-OMe)1]-, [Phe(p-Cl)1]-alloferon, obtained by replacing the His at position 1 with para-substituted phenylalanine derivatives (Czarniewska et al., 2018). The study revealed distinct effects on immune response, highlighting the significance of the first N-terminal His residue of alloferon in influencing the immune characteristics of insects (Czarniewska et al., 2018). The analogues had multiple functions in mealworm, and [Phe (p-(NH_2_)1] -and [Phe (p-OMe)1]- alloferons had the strongest activity evidenced by inhibited cell and humoral defense of *Staphylococcus aureus* infection (Czarniewska et al., 2018). Together, alloferon and the analogues had a proapoptotic effect in the insect.

Generally, it appears that the distinctive biological activity of alloferon analogues, whether cytotoxic or antiviral, is strongly influenced by several factors, including the position of the replaced histidine residue, the specific amino acid residues used as replacements at different positions, and the C- and N-terminal residues. Although knowledge on the antiviral mechanism of the analogues still remains limited, it is evident that the structural elements play a significant role in contributing to the overall activity of the analogues.

## 5 Clinical applications of alloferon and analogues in disease treatment

### 5.1 Combination of alloferon and chemotherapy: enhancing antitumor effects

Chemotherapy is an important part of the multimodal treatment of cancer. Adjuvant chemotherapy after curative resection may significantly improve disease-free survival and overall survival (Springfeld et al., 2019). Numerous preclinical studies have investigated the potential of combining alloferon with chemotherapy to enhance antitumor efficacy and improve outcomes in patients (Bianchi et al., 1994). Alloferon has been shown to improve the delivery of chemotherapeutic agents to tumor sites and enhance immune responses against cancer cells. Gemcitabine (also known as dFdC: 2′,2′-difluorodeoxycytidine), originally used for its antiviral effects (Bianchi et al., 1994), has been widely used as an anti-cancer chemotherapeutic agent for various solid tumors and recently in certain lymphomas as well (Wong et al., 2009). Despite the presence of the studies that used gemcitabine to treat pancreatic cancer, mortality remains significantly higher than in other types of cancer. Therefore, there is a demand for more effective methods to improve conventional PCa therapy.

Alloferon is known to provide support for the low toxicity of gemcitabine in the treatment of patients with advanced PCa, showing high efficacy as an adjuvant in the treatment of PCa (Min et al., 2002). Exposure of Panc-1 and AsPC-1 cells to alloferon increased the chemosensitivity of the cells to gemcitabine, and combined treatment with gemcitabine and alloferon increased apoptosis in Panc-1 cells (Jo et al., 2022). Considering the contribution of alloferon in the gemcitabine chemotherapy for PCa, it might be a useful adjuvant not only for PCa but also for other types of cancer.

More than 500,000 women receive a cervical cancer (CC) diagnosis each year, and CC remains a significant challenge for healthcare around the world. The prevailing method for treating locally advanced cervical cancer (LACC) involves chemotherapy and radiation. Recent research, however, points to innovative and intricate CC treatment strategies that incorporate a range of induction chemotherapies (Todo and Watari, 2016). The anticancer effect of alloferon was examined in research to assess its potential for enhancing the short- and long-term treatment outcomes in patients with LACC through modified neoadjuvant polychemotherapy (NAPCT) (Menshenina et al., 2021). The study involved 237 patients with T2a-3aN0-1M0 squamous cell LACC at the age of 24–61 years and was treated with NAPCT courses. For a greater number of LACC patients, alloferon plus NAPCT in neoadjuvant immunotherapy resulted in tumor elimination in a shorter amount of time, improving overall patient survival and preventing relapses (Menshenina et al., 2021). Moderate antitumor activity of alloferon in DBA/2 mice grafted with P388 murine leukemia cells has also been demonstrated. A pulse immunochemotherapy combination of chemotherapy and alloferon showed enhanced antitumor activity compared to the conventional cytostatic chemotherapy (a mixture of cyclophosphamide, doxorubicin, and vincristine), as well as alloferon monotherapy. Alloferon aided NK cells activation, causing release of a considerable amount of IFN-γ and TNF-α *in vivo*. Furthermore, NK cells were found to have regulatory functions to limit and prevent autoimmunity via killing autologous immune cells (Chernysh et al., 2012). This indicates that alloferon can display antitumor activity in a combination therapy via ameliorating NK cell cytotoxicity, IFN-γ and TNF-α synthesis, and perforin secretion. Thus, alloferon is of interest as a potential anticancer drug, particularly for prevention or treatment of early stages of cancer development when the population of tumor cells is minimal and sensitive to immunological surveillance.

Altogether, alloferon and its analogues possess promising potential as novel antibacterial agents for the treatment of infections. These analogues may contribute to tacking the growing challenge of antibiotic resistance by targeting specific types of microbes or enhancing alloferon’s activity against a broad spectrum of microorganisms. The consideration of alloferon and its analogues provides an additional avenue for researchers interested in insect immune systems, alongside their potential therapeutic applications (da Mata É et al., 2017). The study of alloferon and other AMPs can help to further evaluate the intricate interactions between insects and pathogens, leveraging insects’ immune systems as essential models for understanding the evolution of immunological defense mechanisms. Moreover, conducting more extensive and detailed studies of alloferon on various organisms, beyond insects, including *in vitro* studies or cells, will expand our knowledge and comprehension of the peptide and can pave a way for the development of innovative and more effective treatments for a variety of infections.

### 5.2 Clinical applications of alloferon and their analogues

Alloferon and its analogues has been the subject of numerous clinical studies to that aimed at assessing the AMPs’ effectiveness and safety in humans. Several studies utilized alloferon as the active ingredient in antiviral drugs to treat various viral infections. In a study conducted by Tapilskaya et al., alloferon (Allokin-alpha) was evaluated in the complex treatment of virus-associated chronic endometritis (CE) in 33 women with infertility, papillomavirus infection (PVI) persisting in the uterine cavity, and recurrent herpes-virus infection (Tapilskaya et al., 2022). The administration of alloferon and valacyclovir for 30 days resulted in elimination of HPV and lessened the severity of chronic endometritis (Tapilskaya et al., 2022). Alloferon’s clinical effects were also assessed in 59 patients with chronic Epstein-Barr virus infection (CEBVI) using Allokin-alpha. The treat resulted in reduction of EBV DNA and improvement in clinical complaints for 59.67% of patients. Allokin-alpha worked by improving the recognition of virus-infected cells and suppressing viral replication (Rakitianskaya et al., 2019a). Based on these clinical studies it is recommended that Allokin-alpha therapy can be for the treatment of chronic EBVI at a dose of 1 mg subcutaneously every other day with a course dose of at least 9 injections (Rakitianskaya et al., 2019a). Moreover, allokin-alpha demonstrated its efficacy in treating chronic fatigue syndrome in the presence of chronic herpesvirus infection. A total of 53 patients with chronic fatigue syndrome in the presence of chronic herpesvirus infection were examined. The patients received treatment of allokin-alpha therapy with 9 subcutaneous injections of 1.0 mg every other day (Rakitianskaya et al., 2019b). Administration of allokin-alpha decreased the HHV-6 and EBV DNA and maintained the serum and spontaneous production of IFN-α throughout the period of the therapy (Rakitianskaya et al., 2019b). In a study, 67 patients aged 18–45 years suffering from chronic prostatitis, chronic prostatovesiculitis, chronic uretroprostatitis complicated by excretory-toxic infertility were treated with allokin-alpha (Akimov et al., 2013). The combination of the conventional enzyme therapy with allokin-alpha promoted a more rapid and complete eradication of sexually transmitted infection (STI) pathogens, and normalization of the spermogram in the infected patients (Akimov et al., 2013). In a pilot study, alloferon was combined with pyrogenal and cytokine therapy to treat patients with metabolic syndrome. Complex therapy involving alloferon resulted in activation of cell-mediated mechanisms of adaptive immunity and positive changes in lipid profile. Both alloferon monotherapy and complex therapy involving alloferon caused positive dynamics in the main markers of the lipid profile and a decrease in the level of serum markers of systemic inflammation in the patients with metabolic syndrome (Didkovsky et al., 2019). Overall, these clinical studies indicate that alloferon and its analogues are safe and potentially effective in treating a range of conditions. However, further studies are needed to select the optimal dose and regimen and to further evaluate its efficacy in a larger patient population. The results of the different studies and applications of alloferon are listed in Table 2.

**TABLE 2:** Clinical applications of alloferon.

Disease	Therapy	Mode of action and biological effect	References
Pancreatic cancer (PCa)	Combined therapy with gemcitabine	As an adjuvant, increased the chemosensitivity of cells to chemotherapy drug, apoptosis of infected cells	Min et al. (2002)
Locally advanced cervical cancer (LACC)	Combined therapy with neoadjuvant polychemotherapy (NAPCT)	Rapid tumor elimination, prevents relapses	Menshenina et al. (2021)
Leukemia	Immunochemotherapy of alloferon and chemotherapy	Improved antitumor activity over conventional cytostatic chemotherapy, release IFN-γ and TNF-α, prevents autoimmunity	Chernysh et al. (2012)
Chronic endometritis	Combined therapy with valacyclovir	Eliminates HPV, lessens severity of chronic endometritis	Tapilskaya et al. (2022)
Chronic Epstein-Barr Virus Infection (CEBVI)	Allokin-alpha	Reduces DNA EBV, improves recognition of virus-infected cells, suppresses viral replication	Rakitianskaya et al. (2019a)
Chronic fatigue syndrome	Allokin-alpha	Decreases HHV-6 and EBV DNA, produces interferon	Rakitianskaya et al. (2019b)
Genitourinary infections	Combination of conventional enzyme therapy with allokin-alpha	Rapid and complete eradication of STI pathogens	Akimov et al. (2013)
Metabolic syndrome	Alloferon monotherapy, combined therapy with pyrogenal and cytokine	Positive changes in lipid profile, reduced systemic inflammation	Didkovsky et al. (2019)

However, the current knowledge on alloferon is still insufficient, and more research is needed to fully grasp its potential benefits and drawbacks. The results of additional preclinical and clinical studies, as well as regulatory approvals from health authorities, will determine the future direction of alloferons’ usage. In case of a positive feedback, alloferon may become a strong alternative to conventional antibiotics, antiviral and antitumoral preparations, as well as industrial preservatives in combating diseases and food spoilage. Alloferon possesses a promising potential for succeeding in the areas of food safety and global health.

## 6 Conclusion and perspectives

In our review, we sought to discuss and bring to light the mechanisms underlying the anticancer and antiviral activity of the antimicrobial peptide alloferon, its analogues and also the drugs already used in clinical applications. We discovered that indeed this peptide has powerful therapeutic effects against different viral infections and cancer cells. The NF-κB pathway and NK cell pathway were prominently implicated in the various studies done in the past, and different conclusions reached by diverse reports confirm the involvement of these pathways in the overall effects of the peptide. NF-κB regulation of apoptosis varies, being apoptotic or proapoptotic depending on the cell type and stimuli, which gives understanding to why alloferon activated the NF-κB in certain viral infections while inhibiting the pathway in others. Additionally, we found that the His residue of alloferon affects the biological effects of the drugs, as evidenced by synthetic analogues modified at different amino acid residues exhibiting higher antiviral/antitumor activities over others. The peptide has also been employed as the main active ingredients in drugs and combined with other chemotherapy drugs to enhance their potential and effects in disease treatment.

Alloferon, a novel immunomodulatory peptide, holds promise in many fields such as immunomodulation, antimicrobial therapy, wound healing, and food protection. Studies have shown that alloferon have potent immunomodulatory activity by increasing the production of cytokines and stimulating immune cells, forming the basis for its therapeutic, biological and immunological effects. In regards to its immunomodulatory effect, it is important also to note that it is not limited to only suppression neither is it limited to only activation of immune responses but its role is dependent on the disease setting and peculiar to conditions involved. For example, alloferon may act as an inhibitor of proinflammatory cytokines to suppress inflammation in an autoimmune disease or infection, whereas in diseases like viral infections, it may aid activation of the inflammatory mediators. In addition, a multitude of studies suggest alloferons’ potential to be used as an alternative to synthetic antibiotics due to demonstrated potent antibacterial activity against a wide range of pathogenic microorganisms and in the treatment of various viral infections and cancers. Alloferon, whether applied separately or in combination with other drugs, can exert its antiviral and antitumor effects primarily by activating NK cells. NK cells’ stimulation results in the expression of cytotoxic and proinflammatory cytokines, enzymes, and other receptors. Alloferons also function as interferon mimetics, causing macrophage activation and improving immunological responses by promoting cytokine production. In addition, it has been proposed that alloferon can be used in the food and skin care industries as a natural alternative to industrially produced food preservatives and antioxidants. To summarize, alloferon possesses broad-spectrum antibacterial and antitumor activities along with potent immunomodulatory and anticancer properties. Alloferon and its analogues exhibit potent cytocidal effects against viruses and tumor cells. Therefore, their potential to significantly mitigate the issues of antibiotic resistance in bacterial and chemoresistance in cancer cells is substantial. On the other hand, drawbacks such as poor stability and absorption, limited clinical data, high cost, potential immunogenicity can limit clinical development. The emergence of new technologies will enable the conversion of natural alloferons or the synthesis of new alloferons with desirable properties. Overall, however, alloferon and its analogues have potential uses that hold promise for further research and development as natural immunomodulatory and antimicrobial agents.
